# Fitness for purpose of stabilized stool samples for bile acid metabolite analyses

**DOI:** 10.1038/s41598-021-86784-0

**Published:** 2021-04-12

**Authors:** Lorie Neuberger-Castillo, Wim Ammerlaan, Fay Betsou

**Affiliations:** 1 Integrated BioBank of Luxembourg (IBBL), 1, Rue Louis Rech, L-3555 Dudelange, Luxembourg; 2 Laboratoire National de Sante (LNS), 1, Rue Louis Rech, L-3555 Dudelange, Luxembourg

**Keywords:** Metabolomics, Applied microbiology

## Abstract

Biobanks and cohort studies are increasingly utilizing chemical stabilizers to collect and store stool samples for downstream DNA-based microbiome analyses. While stabilizers permit ambient-temperature collection and storage of samples for gut microbiome studies, the use of the same sample type for downstream metabolomics assays has not been explored. Microbiome-metabolomics analysis of fecal samples is increasingly getting attention to further elucidate the mechanisms by which the gut microbiota influences the host. In this study, we evaluated fitness-for-purpose of OMNIgene-GUT-collected stool samples for downstream metabolomics assays in the scope of fecal bile acids (BA) quantification. Biocrates Bile Acids Kit was used for the quantification of BA from eight healthy donors’ samples collected in (1) OMNIgene-GUT kit and (2) snap frozen in −80 °C in duplicates. A highly selective reversed phase LC–MS/MS analysis method in negative ion multiple reaction monitoring (MRM) detection mode was applied to determine the BA concentrations in each sample.Total fecal BA levels were detectable in OMNIgene-GUT-collected samples (range: 29.9–903.7 pmol/mg). Paired t-test confirmed that there was a significant difference in the total BAs between the OMNIgene-GUT and snap frozen samples (p < 0.05). Extractions from snap frozen samples resulted in higher concentrations of total BAs (range: 243.7–1136.2 pmol/mg). Qualitative differences between individual donors’ BA profiles were detectable using the two sample collection methods. No significant difference was found in the relative concentrations of primary (CA, CDCA) or secondary (DCA, LCA, UDCA) unconjugated BAs to the total BA concentrations in OMNIgene-GUT-collected samples as compared with the snap frozen samples (Wilcoxon-Mann–Whitney test, p > 0.05). Passing-Bablok method comparison and correlation analyis showed a high degree of correlation in the relative concentrations of CA, CDCA, DCA and LCA between OMNIgene-GUT and snap frozen samples. For these four bile acids, the two methods are comparable at an acceptability bias of 30%. We conclude that the OMNIgene-GUT-collected stool samples are fit-for-purpose for downstream fecal bile acids analysis.

## Introduction

Previous studies have established a bidirectional relationship between the host bile acid homeostasis and the gut microbiota, and bacterial overgrowth has been associated with intestinal inflammation and reduced bile acid concentrations in the gut^1–3^. Bile acids (BAs) are natural products of cholesterol synthesis that aid in the emulsification and absorption of dietary fats in the small intestine and are considered to be as important as hormones^4^. The primary BAs, cholic acid (CA) and chenodeoxycholic acid (CDCA), are synthesized in the liver and largely conjugated with glycine and taurine. After performing their critically important function of promoting intestinal fat absorption, approximately 95% are absorbed by ileal BA transporters for recycling back to the liver. The remaining 5% pass to the colon where most undergo other microbiota-mediated biotransformations (e.g. 7-alpha-dehydroxylation from primary to secondary bile acids). Only a small amount of secondary bile acids are reabsorbed by passive diffusion, most is excreted in faeces^5^.

The gut microbiota can change the amount and composition of the BA pool through their effects on BA metabolism, specifically in synthesis, deconjugation and conversion of primary to secondary BA^1^. The quantification of fecal bile acids and the profiling of the gut microbial community are increasingly getting attention to further elucidate the mechanisms by which the gut microbiota influences the host^3,5^. One study which evaluated adults with non-alcoholic fatty liver disease (NAFLD) concluded that gut microbiota dysbiosis is associated with altered bile acid (BA) homeostasis, which renders the patients at increased risk of hepatic injury^6^.

To be able to make meaningful statements about the correlation between the BA homeostasis and gut microbiota composition, it is essential that the same stool sample be used for both analyses. The collection method, which is considered as the gold standard method for downstream analysis of metabolites and the gut microbiome composition, is the immediate freezing of stool samples^7,8^. Previous studies showed that freezing samples in − 20 °C or lower immediately after collection preserves the microbial composition similar to analysis of a fresh sample and also avoids potential influence of added preservative. While preserving the microbial composition, it also preserves the detectability of metabolites^7,8^.

However, this approach is not always feasible in large-scale, population-based studies, home collection and collection in remote areas because of the high costs associated with cold-chain shipping requirements.

On the other hand, biobanks and large-cohort studies usually collect stabilized stool samples with collection kits, such as the OMNIgene-GUT kit, which has extensively been validated for downstream DNA-based microbiome analyses^9^. As per the manufacturer, the mechanism of stabilization is through inhibition of microbial growth and DNA degradation^10^.

While such a kit permits ambient-temperature collection and storage of samples for gut microbiome studies, the use of the same sample type for downstream targeted bile acid quantitative assays has not been explored. Integrated microbiome-metabolomics analyses of fecal samples are increasingly getting attention to further elucidate the mechanisms by which the gut microbiota influences the host. Therefore in this study, we evaluated the fitness-for-purpose of the OMNIgene-GUT-collected stool samples for downstream metabolomics assays in the scope of the fecal bile acids (BA) quantification.

## Results

**Figure 1:**
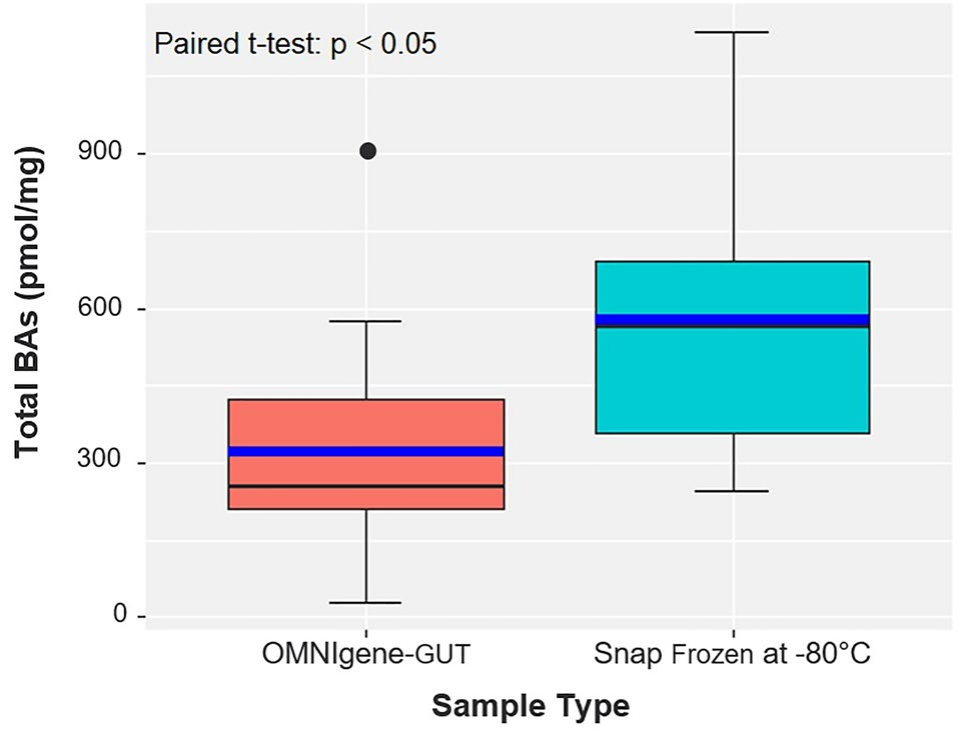
Total fecal bile acid concentrations (pmol) per mg stool, measured from human stool samples stabilized in OMNIgene-GUT and Snap frozen at − 80 °C. The box/whiskers represent the following: 1st Quartile, Median, 3rd Quartile. The blue line represents the Mean. The error bars represent the standard deviation. Paired t-test, p = 0.0029.

**Figure 2:**
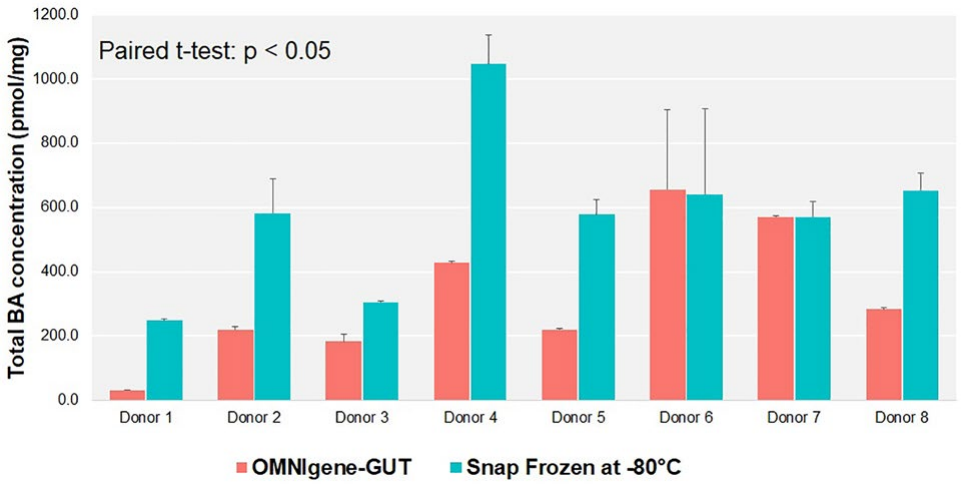
Quantification of endogenous bile acids, measured from eight human stool samples of different mass and stabilized in OMNIgene-GUT and Snap frozen at −80 °C. Results are shown in terms of total fecal bile acid concentrations (pmol) per mg stool. Each bar represents the mean value of duplicate measurements per donor. The error bars represent one standard deviation. Paired t-test, p = 0.0029.

The concentration of total fecal BA levels could be measured in OMNIgene-GUT-collected samples (mean: 323.8 pmol/mg; range: 29.9–903.7 pmol/mg) (Figs. 1, 2 and Table 1). Paired t-test confirmed that there was a significant difference in the total BAs between the OMNIgene-GUT and snap frozen samples (p < 0.05). Extractions from snap frozen samples resulted in higher concentrations of total BAs (mean: 578.1 pmol/mg; range: 243.7–1136.2 pmol/mg). This is caused by the fact that the OMNIgene-GUT-collected samples are diluted in stabilizer (approximately 500 mg of sample in 1.5 ml stabilizing liquid), resulting in lower total fecal BA concentrations.

**Table 1:** Bile Acid Concentration (pmol/mg) of each sample tested per donor.

Donor N°	Storage container	Sample ID	Bile acid concentration (pmol/mg)														
			CA	CDCA	DCA	LCA	UDCA	GCA	GCDCA	GDCA	GLCA	GUDCA	TCA	TCDCA	TDCA	TLCA	TUDCA
Donor 1	OMNIgene-GUT	D1-OMNIg-GUT_1	22.92	32.236	23.788	0.6908	0	0.0648	0.478	0	0	0	0.0408	0.0816	0	0	0.0012
		D1-OMNIg-GUT_2	24.12	31.784	2.454	0.9	0	0.0476	0.4244	0	0	0	0.0284	0.0832	0	0	0.002
	Snap frozen at −80 °C	D1-SnapFrozen_1	206	21.2	15.2	0.8184	0	0.0876	0.0708	0	0	0	0.246	0.036	0.0168	0	0
		D1-SnapFrozen_2	213.6	22.12	15.84	0.6676	0	0.1388	0.0748	0	0	0	0.238	0.0376	0.0244	0	0
Donor 2	OMNIgene-GUT	D2-OMNIg-GUT_1	152.4	29	20.56	0.4924	1.652	19.776	27.952	0	0	0.0532	0.4396	0.6764	0	0	0.0156
		D2-OMNIg-GUT_2	166.8	32	22.6	0.6576	1.794	22.348	29.308	0	0	0.0716	0.4568	0.7972	0	0	0.0148
	Snap frozen at −80 °C	D2-SnapFrozen_1	385.6	48	33.32	13.252	36.196	14.224	0.9752	0	0	0.0376	0.446	0.448	0	0	0.034
		D2-SnapFrozen_2	548	78	52.4	0.5364	5.16	20.348	13.344	0	0	0.0544	0.3296	0.4364	0	0	0.044
Donor 3	OMNIgene-GUT	D3-OMNIg-GUT_1	22.076	0.1596	110.4	86	0	10.688	18.092	11.412	0	0.106	18.236	0.9068	0.3808	0	0.062
		D3-OMNIg-GUT_2	16.552	0.1332	91.2	61.6	0	0.9096	10.468	0.4844	0	0.08	11.816	0.6268	0.256	0	0.0372
	Snap frozen at −80 °C	D3-SnapFrozen_1	31.556	0.03	98.4	204	12.944	0.0844	0.5056	0.5636	0.2836	0.0496	0.1656	0.3156	0.3196	0.156	0.046
		D3-SnapFrozen_2	3.552	0	90.4	204	10.176	0.128	0.6068	0.6392	0.2492	0.0984	0.5336	0.5396	0.382	0.108	0.048
Donor 4	OMNIgene-GUT	D4-OMNIg-GUT_1	0.8408	10.116	278.8	148.8	0	0.1452	0.7988	16.992	0	0.062	0.0404	0.0628	0.09	0	0
		D4-OMNIg-GUT_2	0.9276	1.036	265.2	152	0	0.1388	0.8672	17.252	0	0.0356	0.0444	0.0592	0.082	0	0
	Snap frozen at −80 °C	D4-SnapFrozen_1	3.15	7.64	752	365.2	7.2	0.0584	0.1004	0.5824	0.194	0.0344	0	0.0084	0.0588	0	0
		D4-SnapFrozen_2	14.476	2.074	596	351.2	39.876	0.0696	0.1032	0.6424	0.1152	0.0508	0	0.0056	0.042	0	0
Donor 5	OMNIgene-GUT	D5-OMNIg-GUT_1	0.9956	1.134	83.2	134	0	0.8676	1.816	0.9612	0	0.0564	0.1436	0.2168	0.114	0.0124	0.006
		D5-OMNIg-GUT_2	0.9584	10.256	81.2	128	0	0.7504	14.456	0.9028	0	0.056	0.1408	0.2056	0.1	0.0144	0.0064
	Snap frozen at −80 °C	D5-SnapFrozen_1	10.684	32.744	190.8	332	0	0.1704	0.4692	0.5532	0.1468	0.0344	0.1496	0.2536	0.2588	0.0632	0.0084
		D5-SnapFrozen_2	20.968	0.9776	236	383.6	0	0.3192	0.4056	0.6488	0.2052	0.1336	0.662	0.2068	0.3836	0.06	0
Donor 6	OMNIgene-GUT	D6-OMNIg-GUT_1	0.1608	0.2136	206.4	200.8	0	0.2296	0.7376	0.7228	0	0.046	0.0616	0.0884	0.0884	0.026	0
		D6-OMNIg-GUT_2	0.7508	1.364	460	440	0	0.1016	0.124	0.5484	0	0.0924	0.2632	0.0968	0.2508	0.1104	0.0432
	Snap frozen at −80 °C	D6-SnapFrozen_1	0.7364	9.76	468	428	0	0.1264	0.1384	0.4332	0	0.1148	0.1656	0.0584	0.1948	0.1424	0
		D6-SnapFrozen_2	0.7612	0.2624	178.8	193.6	0	0.2248	0.6368	0.6672	0	0.0476	0.0348	0.0776	0.0764	0.0152	0
Donor 7	OMNIgene-GUT	D7-OMNIg-GUT_1	29.356	0.5664	408	157.2	0	0.7192	0.6348	37.236	0	0	0.1648	0.0604	0.2912	0	0
		D7-OMNIg-GUT_2	29.956	0.53	408	150.4	0	0.7264	0.6436	3.848	0	0.0328	0.1876	0.066	0.3076	0	0.0116
	Snap frozen at −80 °C	D7-SnapFrozen_1	12.044	0	408	209.2	0	0.096	0.044	0.7248	0.2196	0	0	0.014	0.1508	0.042	0.0196
		D7-SnapFrozen_2	0.9796	0	331.2	188.4	0	0.1696	0.0948	0.5992	0.1488	0	0.0332	0.0368	0.17	0.0516	0
Donor 8	OMNIgene-GUT	D8-OMNIg-GUT_1	10.076	2.018	134	135.2	0	11.496	1.402	0.796	0	0.1288	0.2836	0.3304	0.1188	0	0.0192
		D8-OMNIg-GUT_2	1.174	2.184	143.2	136.4	0.576	12.972	17.156	1.036	0	0.1432	0.3096	0.4008	0.1412	0	0.0168
	Snap frozen at −80 °C	D8-SnapFrozen_1	14.484	0.5604	338	364	0	0.2664	0.7544	0.5436	0	0.136	0.0936	0.2856	0.1176	0	0.0108
		D8-SnapFrozen_2	26.372	3.206	273.6	317.6	15.684	0.3276	0.4696	0.272	0	0.0908	0.0592	0.12	0.0724	0	0

CA—Cholic Acid; CDCA—Chenodeoxycholic Acid; DCA—Deoxycholic Acid; LCA—Lithocholic Acid; UDCA—Ursodeoxycholic Acid; GCA—Glycocholic Acid; GCDCA- Glycochenodeoxycholic Acid; GDCA—Glycodeoxycholic Acid; GLCA—Glycolithocholic Acid; GUDCA—Glycoursodeoxycholic Acid; TCA—Taurocholic Acid; TCDCA—Taurochenodeoxycholic Acid; TDCA—Taurodeoxycholic Acid; TLCA—Taurolithocholic Acid; TUDCA—Tauroursodeoxycholic Acid.

**Figure 3:**
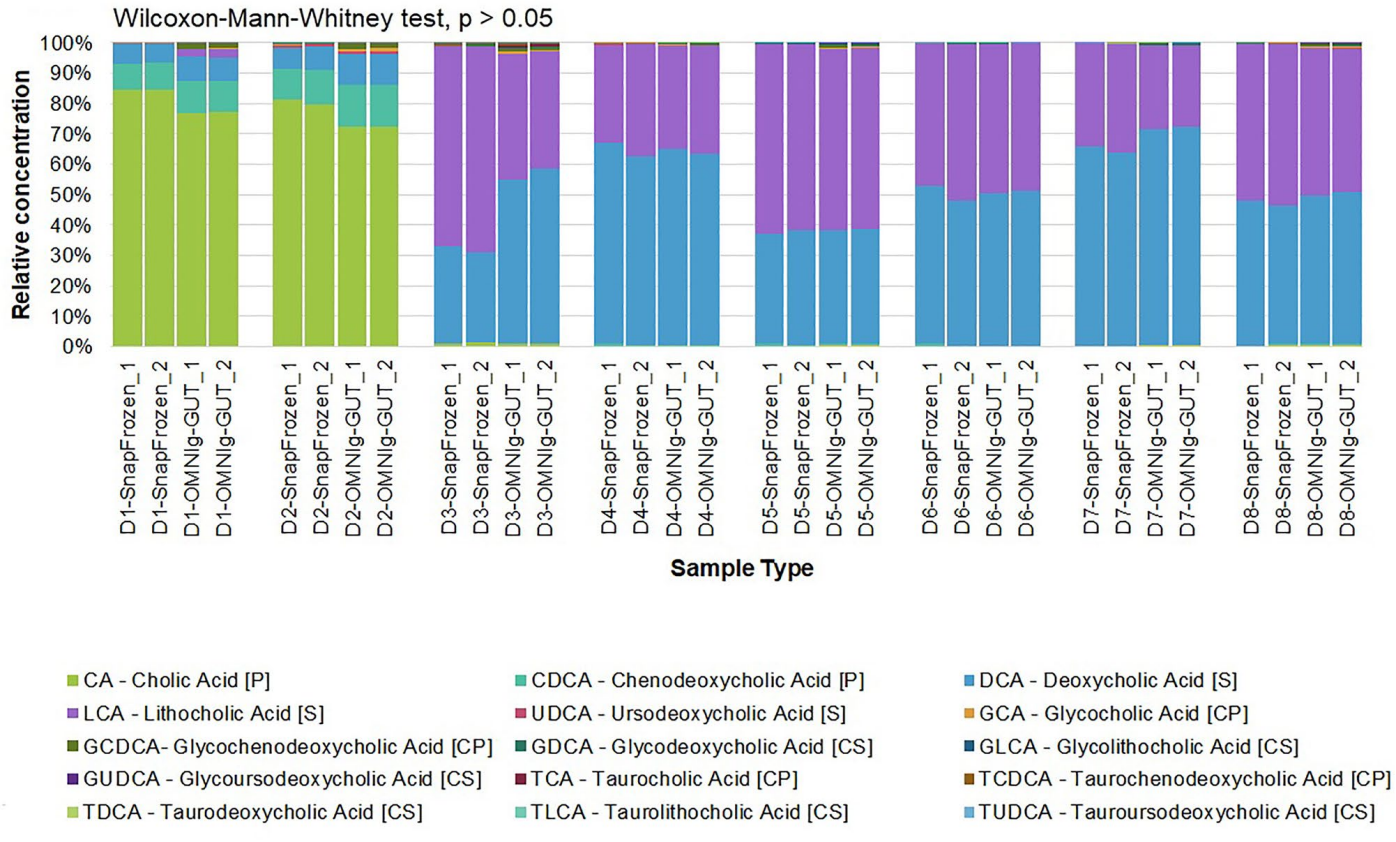
Visual comparison of the relative concentrations of individual bile acids for each sample type (OMNIgene-GUT and Snap frozen at −80 °C) measured in eight donor samples, based on the quantification of bile acids by LC–MS/MS analysis method. (Wilcoxon-Mann–Whitney test, p > 0.05). [P] = primary bile acid; [S] = secondary bile acid; [CP] = conjugated primary bile acid; [CS] = conjugated secondary bile acid.

Variation between each donors’ BA profile is detectable using the two sample collection methods (e.g. the BA profiles of Donors 1 and 2, which are samples collected from children under the age of 2, are readily differentiated among the rest of the donors) (Fig. 3).

No significant difference was found in the relative concetrations of primary unconjugated BAs (CA, CDCA) to the total BA concentrations in OMNIgene-GUT-collected samples as compared with the snap frozen samples (Wilcoxon-Mann–Whitney test, p > 0.05) (Table 1 and Fig. 3). The same result was observed with the relative concentrations of secondary unconjugated BAs (DCA, LCA, UDCA) to the total BA concentrations in both sample collection methods.

**Figure 4:**
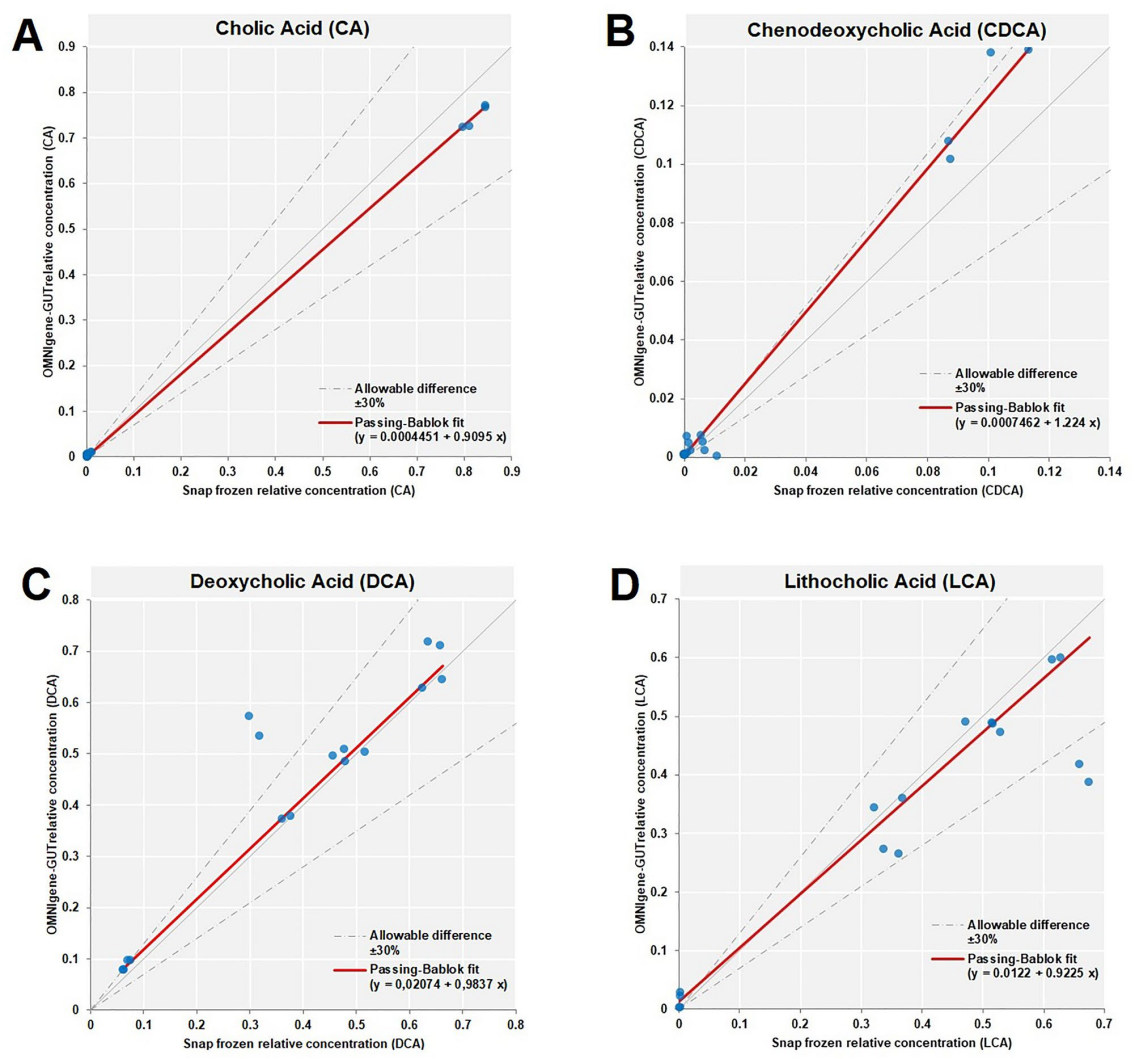
Passing-Bablok method comparison and correlation analysis for bile acids with average relative concentration higher than 0.01. The relative concentrations of Cholic Acid (**A**), Chenodeoxycholic Acid (**B**), Deoxycholic Acid (**C**) and Lithocholic Acid (**D**) in OMNIgene-GUT and snap frozen samples are plotted and indicated by the blue points. The snap frozen is taken as the reference method, while the OMNIgene-GUT is taken as the test method. The Passing-Bablok fit is shown as the red line. The allowable bias between the relative concentrations in OMNIgene-GUT and snap frozen samples is 30%.

To evaluate the degree of correlation in relative concentrations of bile acids between OMNIgene-GUT vs snap frozen samples, we performed Passing-Bablok method comparison and correlation analysis for bile acids with average relative concentration higher than 0.01. Passing-Bablok regression showed a high degree of correlation in the relative concentrations of CA, CDCA, DCA and LCA between OMNIgene-GUT and snap frozen samples (Fig. 4). For these four bile acids, the two methods are comparable at an acceptability bias of 30%.

## Discussion

Excellent reviews of preanalytical aspects of fecal metabolomics have been published^11,12^, highlighting the variability in the protocols used for fecal sample collection, processing (metabolite extraction) and metabolite analysis, the impact of such variations on the recovery of fecal metabolites, and the need for both preanalytical and analytical guidelines. It has specifically been shown that the type of solvent used, as well as the ratio fecal:solvent influence the accuracy of analyses by nuclear magnetic resonance (NMR), while freeze thaw and sonication have less impact^13,14^. In this study, we used a fully validated analytical method [Pham et. al, 2016] that has been optimized by Biocrates for use with plasma samples^15^. This validated protocol was adapted for stool samples using a modified protocol published by the same manufacturer^16^.A study by De Spiegeleer et al. using lyophilized fecal samples, has shown that freeze thawing affects the polar more than the lipid metabolome, with 10% and 7% of metabolites showing statistically significant differences after one freeze thaw cycle^17^. Previous studies have also shown an impact of freeze thawing, probably due to reactivation of microbial biochemical reactions upon thawing, however such changes were small relative to inter-individual differences^18^. In our study, all samples underwent two freeze thaw cycles before analysis, and it can be assumed that the potential bias was the same in all samples.

RNA*later* is a very commonly used stabiliser for nucleic acids in different types of biospecimens, including feces. However, it has been repeatedly shown that RNA*later* is not fit for purpose for any metabolomics analyses, probably due to high sodium sulphate content^7,8,19^. There has been a previous preanalytical study, including OMNIgene-GUT tubes, and comparing them to snap frozen fecal samples, and samples collected in ethanol, RNA*later* or on FTA cards, in the scope of 16S rRNA gene sequencing, and untargeted and targeted metabolomics^7^. This study showed that OMNIgene-GUT stabiliser is suboptimal for untargeted metabolomics analyses, due to lower sensitivity, but fit for purpose for targeted metabolomics on short chain fatty acids (SCFA), with high concordance with the snap frozen samples. A more recent study by Lim et. al. suggested that it is possible to extract both microbiome and metabolite data from a single stool sample collected using OMNIgene-GUT tubes^20^. Our study has also found fitness for purpose of OMNIgene-GUT tubes for targeted metabolomics for another class of metabolites, the bile acids.

We conclude that the OMNIgene-GUT-collected stool samples are fit-for-purpose for downstream metabolomics assays in the scope of quantitative fecal bile acids analysis. However, the reference ranges of the total and individual fecal BA concentrations are different in OMNIgene-GUT-collected samples and in snap frozen samples. Therefore, the values obtained from the two collection methods cannot be compared directly. However, there is a high degree of correlation in the relative concentrations of CA, CDCA, DCA and LCA between OMNIgene-GUT and snap frozen samples.

Finally, at the time of writing of this article, a new stool collection device has been commercialized by DNA Genotek, the OMNImet-GUT stool collection device, which is declared by the manufacturer to be specifically fit for purpose for metabolomics analyses.

## Methods

### Sample collection

For the comparison of snap frozen and OMNIgene-GUT-collected stool samples in the scope of fecal BA quantification, fresh stool samples were collected by 8 healthy donors in a sterile stool collection container (Sarstedt, ref: 80.734.311) and brought to the Integrated BioBank of Luxembourg (IBBL) laboratory within 2–3 h of collection. This study was approved by the National Research Ethics Committee of Luxembourg (Comité National d'Ethique de Recherche—CNER approval #201,107/02). All experiments and analyses were executed in accordance with the approved guidelines and relevant regulations, and informed consent was obtained from each participant.

Upon arrival in the laboratory, each fresh donor sample was then aliquoted into two separate containers: the OMNIgene-GUT kit (DNA Genotek, ref: OMR-200) and plain 2 ml cryovial (Greiner, ref: 126,263-2D1). For the OMNIgene-GUT kit, approximately 500 mg of fresh sample was placed in the tube containing the stabilizer. The tube was homogenized by mixing and stored temporarily at room temperature (RT) for 3–5 days to mimic RT transport conditions. After storage at RT, the sample in the OMNIgene-GUT tube was aliquoted into two 2-ml cryovial containing 1 ml of stool suspension each and frozen at −80 °C for two weeks.

For the snap frozen sample, approximately 1 g of the fresh donor sample was placed in a plain 2 ml cryovial. This cryovial was frozen immediately in −80 °C for two weeks.

### Sample preparation

Eight OMNIgene-GUT-stabilized samples and eight snap frozen samples were thawed and each sample was further aliquoted into another cryovial. The weight of each sample was recorded prior to re-freezing. A total of 32 frozen stool samples were sent to Biocrates Life Sciences AG(Innsbruck, Austria) for the quantification of endogenous bile acids. See Table 2 for sample details.

**Table 2:** Details of 32 samples used for the quantification of endogenous bile acids.

Donor N°	Storage Container	Sample ID	Amount used for fecal BA quantification (weight in grams)
Donor 1	OMNIgene-GUT	D1-OMNIg-GUT_1	0.6503
		D1-OMNIg-GUT_2	0.2906
	Snap frozen at −80 °C	D1-SnapFrozen_1	0.1993
		D1-SnapFrozen_2	0.1452
Donor 2	OMNIgene-GUT	D2-OMNIg-GUT_1	0.6685
		D2-OMNIg-GUT_2	0.5691
	Snap frozen at −80 °C	D2-SnapFrozen_1	0.5736
		D2-SnapFrozen_2	0.2074
Donor 3	OMNIgene-GUT	D3-OMNIg-GUT_1	0.6368
		D3-OMNIg-GUT_2	0.5140
	Snap frozen at −80 °C	D3-SnapFrozen_1	0.2304
		D3-SnapFrozen_2	0.1988
Donor 4	OMNIgene-GUT	D4-OMNIg-GUT_1	0.8189
		D4-OMNIg-GUT_2	0.3327
	Snap frozen at −80 °C	D4-SnapFrozen_1	0.1878
		D4-SnapFrozen_2	0.1931
Donor 5	OMNIgene-GUT	D5-OMNIg-GUT_1	0.7210
		D5-OMNIg-GUT_2	0.4504
	Snap frozen at −80 °C	D5-SnapFrozen_1	0.1989
		D5-SnapFrozen_2	0.1560
Donor 6	OMNIgene-GUT	D6-OMNIg-GUT_1	0.8290
		D6-OMNIg-GUT_2	0.3123
	Snap frozen at −80 °C	D6-SnapFrozen_1	0.3690
		D6-SnapFrozen_2	0.2228
Donor 7	OMNIgene-GUT	D7-OMNIg-GUT_1	0.8288
		D7-OMNIg-GUT_2	0.3430
	Snap frozen at −80 °C	D7-SnapFrozen_1	0.5330
		D7-SnapFrozen_2	0.2908
Donor 8	OMNIgene-GUT	D8-OMNIg-GUT_1	0.7669
		D8-OMNIg-GUT_2	0.4655
	Snap frozen at −80 °C	D8-SnapFrozen_1	0.2328
		D8-SnapFrozen_2	0.1822

To extract metabolites from feces, threefold volume of ethanol/phosphate buffer (85:15 v/v) was added to each stool sample and was vortexed for 3 min. The homogenized samples were then placed in a plate shaker for 30 min at 200 rpm at 0 °C. Following this step, the samples were sonicated at 70 W for 5 min at 0 °C and centrifuged at 800 g for 10 min at 0 °C. The supernatant from each tube was then transferred into a new reaction tube and centrifuged at 19,000 g for 10 min at 4 °C. The new supernatants from this step were transferred into new reaction tubes and were used for analysis. 10µL of supernatant/fecal extracts were used for the Biocrates’ Bile Acids Kit according to the manufacturers’ instructions.

### Quantification of fecal BA

The endogenous bile acids were quantified in collaboration with the Metabolic Phenotyping Services Center of Biocrates Life Sciences AG, (Innsbruck, Austria) using a mass spectrometric-based metabolomic approach with the commercially-available Biocrates’ Bile Acids Kit^21^. A highly selective reversed phase LC–MS/MS analysis method in negative ion multiple reaction monitoring (MRM) detection mode was applied to determine the concentrations of bile acids. The samples were extracted via dried filter spot technique in 96-well plate format. Sample extracts were measured by LC–ESI–MS/MS with a tandem mass spectrometry instrument (TSQ Vantage, Thermo Fisher Scientific TSQ). For highly accurate quantification, 7-point external calibration curves and 10 stable isotope-labeled internal standards were applied. Data of bile acids were quantified using the appropriate MS software (Thermo Fisher Scientific Xcalibur) and the results were finally imported into Biocrates MetIDQ software for further analysis.

Accuracy of the measurements (determined with the accuracy of the calibrators) was in the normal range of the method (deviations from target ≤ 20%) for all analytes. Quality control samples were within the pre-defined tolerances of the method.

### Statistical analyses

All statistical analyses were conducted with Analyse-IT Method Validation Edition, v.4.60.01 (Analyse-it Software, Ltd.) and Microsoft Office Excel 2016. The analysis of distribution of total BAs data was done by performing Shapiro–Wilk test. Paired t-test was performed to compare the total BAs in both OMNIgene-GUT and snap frozen samples. The comparison between the relative concentrations of primary BAs (CA, CDCA) and secondary BAs (DCA, LCA, UDCA) to the total BA concentrations in OMNIgene-GUT-collected samples and snap frozen samples were conducted using the Wilcoxon-Mann–Whitney test. For bile acids with an average relative concentration higher than 0.01 (CA, CDCA, DCA and LCA), Passing-Bablok method comparison and correlation analysis was performed. For all the tests performed, the statistical significance was accepted at p < 0.05.
